# Accurate measurements of slice electron beam parameters at the undulator in seeded free-electron lasers

**DOI:** 10.1107/S1600577524011585

**Published:** 2025-01-01

**Authors:** Enrico Allaria, Paolo Cinquegrana, Miltcho B. Danailov, Eugenio Ferrari, Fabian Pannek, Giuseppe Penco, Eleonore Roussel, Carlo Spezzani

**Affiliations:** a Elettra-Sincrotrone Trieste SCpA 34149Basovizza Trieste Italy; b Deutsches Elektronen-Synchrotron Notkestraße 85 22607Hamburg Germany; c Institute for Experimental Physics University of Hamburg Luruper Chaussee 149 22761Hamburg Germany; d Univ. Lille CNRS UMR 8523 – PhLAM – Physique des Lasers Atomes et Molécules F-59000Lille France; SESAME, Jordan

**Keywords:** slice parameters, energy spread, seeded FEL, free-electron lasers

## Abstract

The operation of modern free-electron lasers (FELs) necessitates precise knowledge of electron beam properties at the undulator to ensure the level of control required by increasingly demanding experiments. In seeded FELs, where only electrons interacting with the seed laser contribute to the process, it is crucial to determine the local values of these properties. We present a novel method, based on accurate modeling of the FEL process in high-gain harmonic generation, to accurately retrieve the electron beam slice energy spread, current and laser-induced energy modulation. Understanding these values is essential for enabling advanced FEL schemes and optimally setting advanced seeding schemes such as echo-enabled harmonic generation. We describe the method and provide an experimental application to the FERMI FEL-1, where a slice energy spread in the range 40–100 keV with a few keV accuracy is measured.

## Abstract 2

A new method has been developed for accurately measuring the slice electron beam current, energy spread, and seed-induced energy modulation in seeded free-electron lasers (FELs). Determining these parameters is crucial for advanced FEL operations and modern seeding schemes like echo-enabled harmonic generation (EEHG). Consequently, this method has become an essential tool for the upgraded FERMI FEL-1.

## 1. Introduction

Free-electron lasers (FELs) (Saldin *et al.*, 2000 ▸) are powerful scientific instruments that have undergone significant evolution in recent decades. Since the initial proposal of FEL amplification in an undulator (Madey, 1971 ▸), numerous laboratories have utilized infrared (IR) and visible FELs in various configurations to advance scientific research (Marangos, 2011 ▸). At shorter wavelengths, following the first demonstration of high-gain amplification in a self-amplified spontaneous emission (SASE) FEL (Milton *et al.*, 2001 ▸), the first user facility was established in the extreme ultraviolet (EUV) range (Ackermann *et al.*, 2007 ▸), succeeded by large-scale facilities delivering powerful radiation down to hard X-rays (McNeil & Thompson, 2010 ▸).

The rapid expansion of user-driven experiments at FELs (Bostedt *et al.*, 2016 ▸) has led to evolving demands for photon properties, driven by the inherent flexibility of FELs. Over the years, several specialized configurations of FELs have been implemented, enabling novel experimental techniques. Today, many schemes, initially introduced as special operating modes, such as two-color/two-pulse (Allaria *et al.*, 2013 ▸; Lutman *et al.*, 2014 ▸; Marinelli *et al.*, 2015 ▸; Hara *et al.*, 2013 ▸) or short pulses (Duris *et al.*, 2020 ▸; Mirian *et al.*, 2021 ▸), are routinely available to scientists for specific experiments.

Externally seeded FELs (Yu, 1991 ▸), initially proposed to generate narrow bandwidth and Fourier-limited pulses (Yu *et al.*, 2000 ▸; Allaria *et al.*, 2012 ▸), have also evolved. Several advanced methods have been developed to precisely control radiation properties, including time structure, phase, chirp and polarization (Allaria *et al.*, 2014 ▸; Ribič *et al.*, 2014 ▸; Prince *et al.*, 2016 ▸; Wituschek *et al.*, 2020 ▸).

These extended capabilities of FELs are accompanied by increased setup complexity, necessitating enhanced control of all involved parameters. Consequently, the ability to accurately measure the properties of the electron and laser beams has become critical to properly configure the parameters required for specific experimental setups.

### 1.1. Electron beam characterization

Modern linear accelerators that drive X-ray FELs are equipped with sophisticated diagnostic tools that enable accurate measurements of electron beam properties. Among these tools, transverse deflectors are particularly important as they allow time-resolved measurements of specific electron beam properties (Emma *et al.*, 2000 ▸).

These deflectors are highly efficient in measuring the current profile along the electron beam and, when used in conjunction with a dispersive element, can also measure the energy distribution of the electron beam (Alesini *et al.*, 2006 ▸). However, as described by Craievich *et al.* (2015 ▸), RF deflectors induce an additional slice energy spread proportional to the deflecting voltage that could be in principle taken into account. However, when the induced slice energy spread is comparable with the intrinsic slice energy spread the accuracy of the measurements is strongly degraded. Moreover, the r.m.s. energy spread resolution (σ_E,res_) at the spectrometer screen depends on the optics function at the screen (beta function, geometric emittance and dispersion) and on the screen resolution itself: for the FERMI case, we have estimated a σ_E,res_ of about 50 keV at 1.2 GeV. Finally, these diagnostic setups are often only available at certain locations within the accelerator, limiting the ability to directly measure beam properties within the FEL undulators. This constraint hampers the accurate prediction of the output FEL properties.

For seeded FELs, the slice energy spread is a crucial parameter that influences the FEL performance especially at high harmonics, as depicted in the following. Various methods have been proposed to measure the slice energy spread of the electron beam, either through the use of deflectors or by indirectly observing the properties of the radiation emitted by the beam in an undulator. The optical klystron effect has proven to be an effective method for determining a slice energy spread averaged along the longitudinal portion of the portion of the beam contributing to the SASE emission (Saldin *et al.*, 2005 ▸; Penco *et al.*, 2015 ▸; Prat *et al.*, 2017 ▸; Prat *et al.*, 2024 ▸; Geloni *et al.*, 2021 ▸). Additionally, for seeded FELs, it has been demonstrated that the slice energy spread can be measured by observing the FEL intensity as a function of the seeded laser power and chicane dispersion (Feng *et al.*, 2011 ▸).

## 2. Method

In this work, we introduce a novel method that leverages the capabilities of numerical codes (Reiche, 1999 ▸) and sophisticated models (Pannek *et al.*, 2023 ▸) to predict FEL performance and accurately reproduce the sensitivity of the FEL to parameter variations. This method is particularly suitable for externally seeded FELs, specifically high-gain harmonic generation (HGHG) FELs (Yu, 1991 ▸), with a seed laser shorter than the electron beam.

By measuring the FEL response to variations in one of the seeding parameters, we can extract the values of the beam current and energy spread in the region where the seed laser interacts with the beam. This is achieved by identifying the parameters that best match the experimental data with the model. Additionally, the method provides insights into the strength of the energy modulation introduced by the seeded laser and any residual chirp.

### 2.1. Bunching and FEL in HGHG

**Figure 1:**
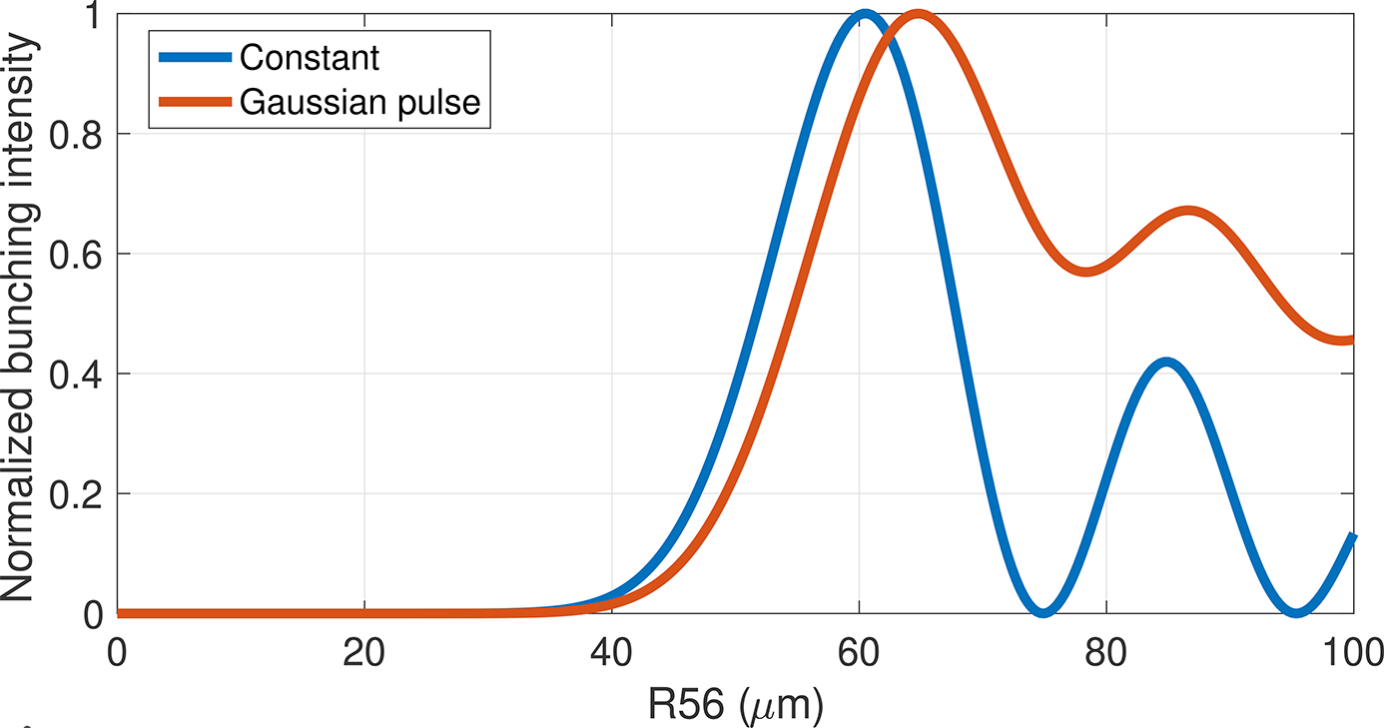
Normalized bunching intensity (obtained as the integral of 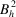 over the pulse length) as a function of *R*_56_ for typical parameter values, assuming a constant energy modulation Δ_γ_ (blue curve) and with a Gaussian temporal profile (red curve).

The FEL process in HGHG is driven by the bunching (*B*) created by converting the coherent energy modulation (Δ_γ_) introduced by the laser in the modulator through the dispersion (*R*_56_) in a chicane. This bunching at the desired harmonic (*h*) of the seed laser wavelength (λ_seed_) initiates the FEL amplification process and is well described by the following equation (Hemsing *et al.*, 2014 ▸),
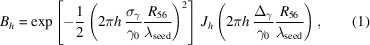
where *J*_*h*_ is the *h*th-order Bessel function of the first kind, while γ_0_ and σ_γ_ are the average beam energy and r.m.s. slice energy spread of the electron beam, respectively. Under standard conditions for HGHG, where Δ_γ_ > *h*σ_γ_, the bunching has a first maximum close to the point where 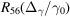 ≃ 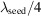. Subsequent maxima follow for larger dispersion (see Fig. 1 ▸, blue curve), with their intensities dominated by the exponential part in equation (1), which strongly depends on the beam energy spread σ_γ_.

When neglecting diffraction and slippage effects along the modulator and assuming a laser waist much larger than the transverse electron beam size, the maximum coherent energy modulation Δ_γ_ introduced by the laser in the undulator of length *L*_u_ can be approximated by (Hemsing *et al.*, 2014 ▸) 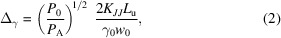
with the peak power *P*_0_ of the seed laser, the constant *P*_A_ = *I*_A_*m*_e_*c*^2^/*e* ≃ 8.7 GW described by the Alfén current *I*_A_ = 4πɛ_0_*m*_e_*c*^3^/*e* ≃ 17 kA, the Bessel modified undulator parameter *K*_*JJ*_, the average beam energy γ_0_ ≫ Δ_γ_, and the 1/*e*^2^-waist *w*_0_ of the laser. The energy of a Gaussian pulse is related to its peak power via 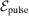 = 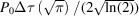, where Δτ denotes the FWHM duration of the pulse.

The final r.m.s. beam energy spread after the modulator can be estimated by 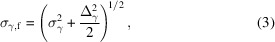
where σ_γ_ is the initial r.m.s. energy spread at the beginning of the modulator.

### 2.2. HGHG sensitivity to *R*_56_

In the case of a seed laser pulse shorter than the electron beam and with a Gaussian temporal profile, the varying amplitude of the induced energy modulation along the electron beam results in local maximum bunching being reached at different values of the dispersion parameter *R*_56_. Consequently, scanning *R*_56_ can significantly alter the FEL pulse profile in both the time and spectral domains (Labat *et al.*, 2009 ▸; Gauthier *et al.*, 2015 ▸). The oscillating features of FEL intensity as a function of dispersion are preserved even though, due to the temporal dependence of the energy modulation, their amplitude is less pronounced with respect to the constant modulation case (Fig. 1 ▸, red curve).

Additionally, the output FEL pulse energy at the radiator exit depends on the amplification occurring in the radiator, which is influenced by the undulator length and the electron beam brightness (primarily the peak current). The entire process can be accurately simulated using the FEL simulation code *Genesis-1.3* (Reiche, 1999 ▸). Recently, a new model has been presented (Pannek *et al.*, 2023 ▸) that is capable of reproducing the pulse energy and the spectral properties of an HGHG FEL with high accuracy and with a significant reduction in terms of computational resources and time with respect to the full simulation.

**Figure 2:**
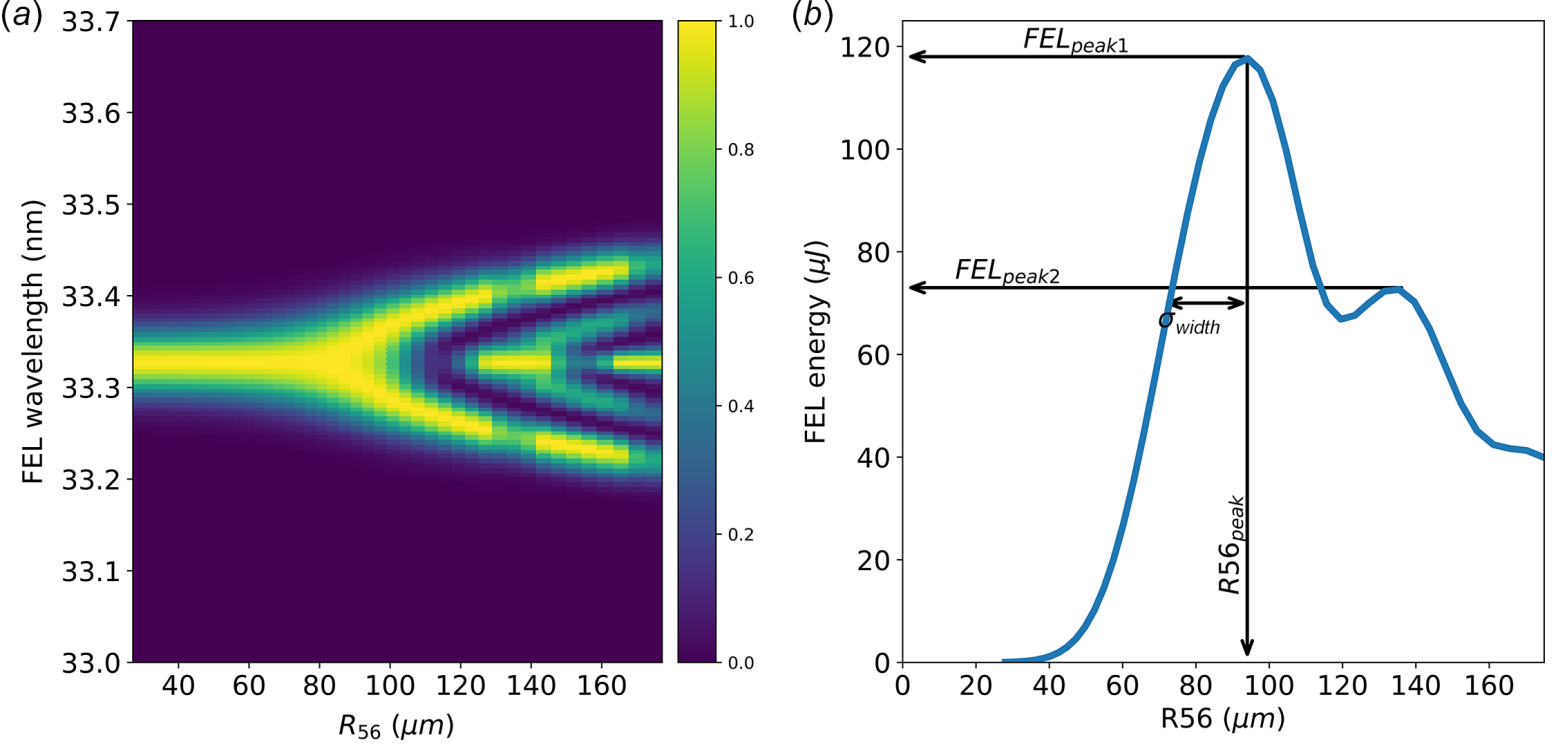
FEL spectra (*a*) and energy (*b*) as a function of *R*_56_ obtained with the model described by Pannek *et al.* (2023 ▸). The following few parameters are defined to characterize the curve: the value of the FEL energy at the first peak (*FEL*_peak1_); the *R*_56_ value giving the maximum FEL energy (*R*56_peak_); the width of the Gaussian fit of the first peak (σ_width_) and the ratio between the FEL energy at the first and second peaks (*R* = 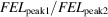).

This allows the model calculations to be included in a sort of fitting procedure enabling the experimental results of the HGHG measurements to be reproduced. Fig. 2 ▸ presents the predicted FEL response to an *R*_56_ scan using such a model with standard FERMI parameters. The calculated FEL spectrum [Fig. 2 ▸(*a*)] and energy per pulse [Fig. 2 ▸(*b*)], which are two of the most easily measured FEL parameters experimentally, are reported. Detailed spectral features have proven useful for determining the chirp in the seed laser and the FEL (Gauthier *et al.*, 2015 ▸; Pannek *et al.*, 2023 ▸). In this work, we focus, instead, on the features in the evolution of the FEL energy per pulse versus *R*_56_ dispersion [Fig. 2 ▸(*b*)].

**Figure 3:**
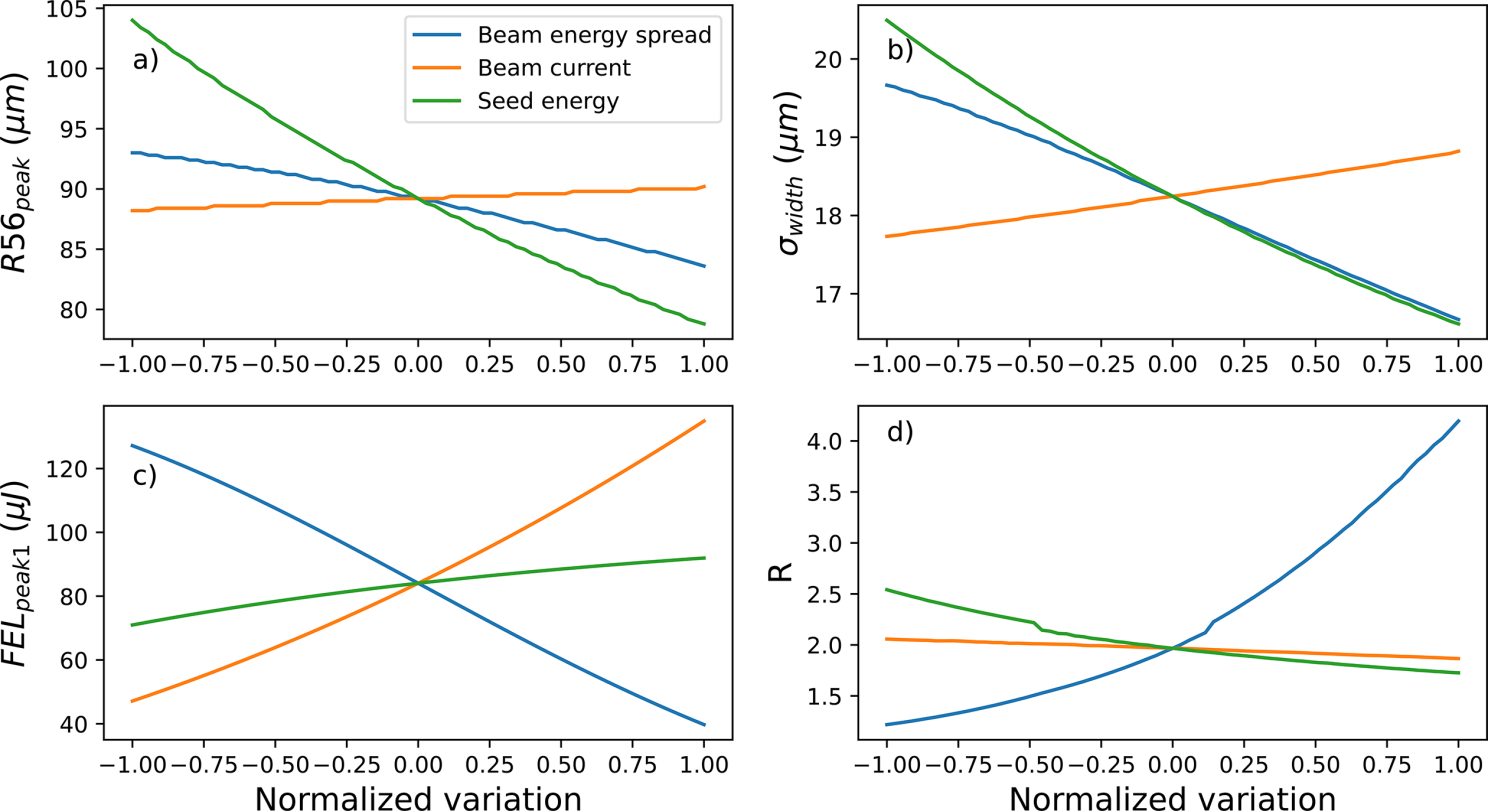
Changes of the defined parameters (*a*) *R*56_peak_, (*b*) *FEL*_peak1_, (*c*) σ_width_ and (*d*) *R* as a function of changes to the electron beam slice energy spread (blue curves), beam peak current (orange curve) and seed laser energy (green curves). Changes to parameters are normalized such that variation = [(value − nominal value)/half range] to simplify the graph. The electron beam current is varied by ±50 A for a nominal current of 550 A, energy spread is varied by ±30 keV for a nominal slice energy spread of 60 keV, and seed laser energy is varied by ±5 µJ for a nominal energy of 20 µJ.

From the FEL pulse energy versus *R*_56_ plot, we can identify several relevant quantities to describe the graph. These include the pulse energy of the FEL at the first peak (*FEL*_peak1_), the *R*_56_ value at the first peak (*R*56_peak1_), the ratio between the FEL pulse energy at the first two peaks (*R* = 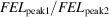), and the width of the first peak (σ_peak_), calculated as the standard deviation of the Gaussian fit of the peak. The impact of variations in the main electron and seed laser parameters, such as beam slice energy spread, current and seed laser energy, on these parameters is reported in Fig. 3 ▸.

The initial value and the range used for each parameter (see details in the caption of Fig. 3 ▸) reflects the nominal value and the expected uncertainty. Within this range, we can distinguish quite different responses to the different parameters. This allows, given an experimental curve of the FEL intensity versus *R*_56_, to almost uniquely identify the values for the three parameters. Indeed, the value of the slice energy spread will mostly be determined by the ratio between the two peaks (*R*), and the seed laser energy will be determined by the dispersion at the FEL maximum (*R*56_peak_); with these two parameters being fixed, the FEL maximum (*FEL*_peak1_) will be determined by the beam current. After a few iterations, one easily converges to the values for current, slice energy spread and energy modulation that best reproduce the experimental results. The non-linear response of *R* to the slice energy spread suggests that the method is suitable for the parameters here considered down to the slice energy spread as low as 30 keV. Conditions for the method to work are those for HGHG (Yu, 1991 ▸) requiring that the energy modulation induced by the seed is larger than the slice energy spread multiplied by the harmonic numbers; if limited in the amount of energy modulation induced by the seed, the method will only be possible at lower harmonics.

## 3. Experimental setup

**Figure 4:**
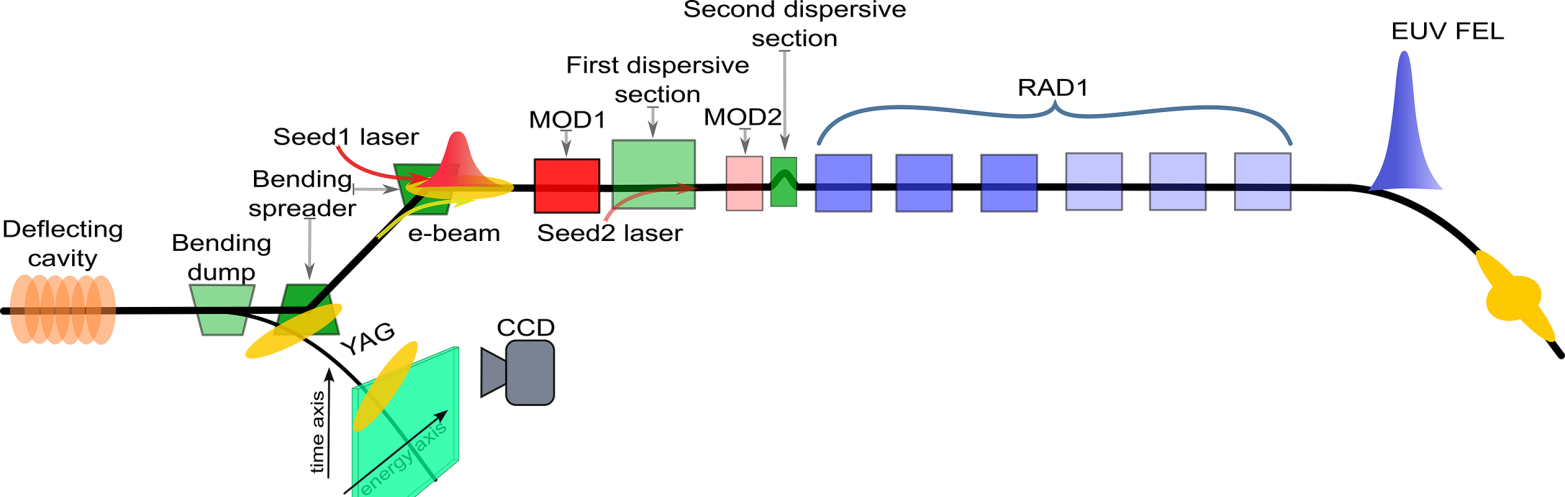
FERMI FEL-1 setup. A deflecting cavity at the end of the linac, in combination with the beam dump, is used to measure the slice properties of the electron beam (Fig. 5 ▸). During FEL operation, the deflection cavity and bending dump are turned off, allowing the beam to enter FEL-1. For the HGHG configuration used for this work, interaction with the laser occurs in the first modulator (Mod1), and bunching is produced in the second dispersive section. Coherent emission is generated at the desired harmonic of the seed laser in the first three undulators of Rad1. The other three undulators are not in resonance.

The measurements were conducted at FERMI’s FEL-1 setup (Allaria *et al.*, 2015 ▸; Allaria *et al.*, 2012 ▸). The FEL-1 line (Fig. 4 ▸) was recently upgraded to echo-enabled harmonic generation (EEHG) (Spezzani *et al.*, 2024 ▸) with the installation of a second modulator (Mod2), a large chicane as the first dispersive section, and a second laser to seed the beam in the second modulator.

It is still possible to run FEL-1 in HGHG mode using either Seed1 or Seed2 (Spezzani *et al.*, 2024 ▸). In both cases, bunching is achieved by appropriately configuring the second dispersive section. When using Seed1, interaction with the electron beam can occur in either the first or the second modulator. If the first modulator is used, the first dispersive section must be set to zero, and the second modulator must be fully open to prevent any degradation of the Seed1-induced energy modulation. When Seed1 interacts with the electron beam in the second modulator, the first dispersive section can be varied over the entire range, provided the timing of the seed laser is adjusted to compensate for variations in the electron beam arrival. If Seed2 is used, the first dispersive section should be set to a relatively high dispersion value (*R*_56_ > 1 mm) that allows the Seed2 injection mirror to be inserted on the electron axis, while the first modulator remains open to avoid interference.

The slice energy spread of the beam can be controlled by adjusting the intensity of the laser heater (Spampinati *et al.*, 2014 ▸), which is installed in the low-energy part of the accelerator. The beam slice energy spread is expected to vary as a function of the square root of the laser heater energy (Saldin *et al.*, 2004 ▸; Huang *et al.*, 2010 ▸).

### 3.1. Electron beam

The FERMI linear accelerator (linac) generates electron beams with energies up to 1.5 GeV, with the nominal parameters used during the reported experiment listed in Table 1 ▸.

**Figure 5:**
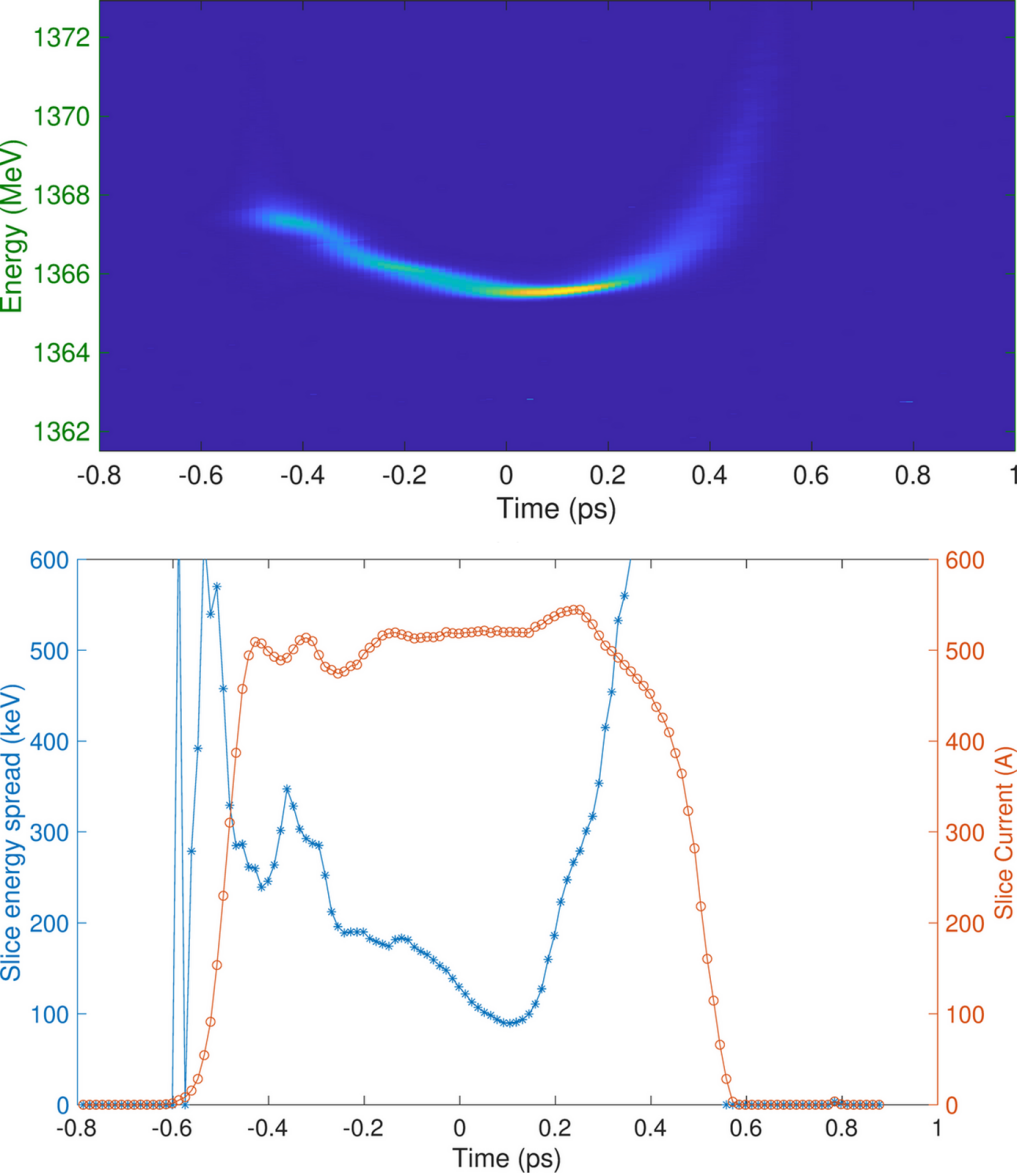
Typical FERMI electron beam longitudinal phase space measured at the end of the linac. The seed laser, typically ∼100 fs long, is normally placed in the flat central region (between −100 and 200 fs).

Energy and current profiles are measured at the end of the linac using dedicated deflector structures (Fig. 5 ▸) (Craievich *et al.*, 2015 ▸). The measured beam phase space also provides information about the beam slice energy spread, which is significantly influenced by the high value of the laser heater and the additional slice energy spread induced by the RF deflector structures. Methods (Prat *et al.*, 2020 ▸) have been proposed and used to deconvolve this effect to determine the natural spread of the beam energy, but they have not been implemented here. In the experimental condition reported here, the minimum slice energy spread that is possible to measure with the RF deflector at FERMI is around 90–100 keV. The emittance is measured in front of the undulator using the quadrupole scan method, and the average beam size is measured using the YAG screen available in each undulator section in the FEL radiator (Bocchetta *et al.*, 2007 ▸).

### 3.2. Seed laser

For the reported experiment the first seed laser has been used on Mod1. The parameters of the used seed laser and their accuracy are provided in Table 2 ▸. Wavelength and bandwidth are measured with high accuracy using a dedicated spectrometer. The pulse length is measured in the laser laboratory prior to the experiment using a frequency-resolved optical gating (FROG) setup, which includes a mockup of the transport line to account for the dispersion of the components encountered by the seed pulse before reaching the interaction point. Energy is measured with a calibrated energy meter near the laser entrance to the vacuum chamber; this measurement may be affected by additional losses at the entrance window, ranging from 5 to a maximum 15% depending on the window state of deterioration. The seed laser spot size at the interaction point is determined from measurements on a dedicated ‘virtual undulator’ setup located on the seed insertion breadboard [see Fig. 8 of Cinquegrana *et al.* (2021 ▸)] where a fraction of the seed beam propagates the same distance and impinges on a fluorescent screen. Due to the non-perfect beam quality confirmed by the value for the laser beam quality factor *M*^2^ (Svelto, 2013 ▸) larger than 1 (*M*^2^ ≃ 1.5), the estimated uncertainty for the spot size at the undulator is approximately 30%.

### 3.3. The HGHG Setup

The FEL parameters are reported in Table 3 ▸ and are obtained from the calibration tables of the devices used. Additional details on the setup can be found in the paper by Kokole *et al.* (2010 ▸).

For this experiment, only three of the six available undulators of the FEL-1 radiator are tuned to the desired harmonic of the seed laser. This prevents the FEL from entering the saturation regime, which may be more challenging to model or simulate. For most of the reported results, the FEL has been operated at the eighth harmonic of the seed laser, resulting in a 33.3 nm wavelength, but measurements at other harmonics have also been conducted.

## 4. Results

The electron beam and the laser with the parameters reported in Tables 1 ▸ and 2 ▸ were used to generate the harmonic *h* = 8 in the first three radiator undulators of FEL-1, tuned at λ = 33.3 nm. After the standard FEL optimization, which includes the laser heater overlap and optimization, seed laser overlap, electron beam matching and undulator resonance adjustments, a scan of the chicane dispersion (*R*_56_) was performed.

### 4.1. Example measurement

**Figure 6:**
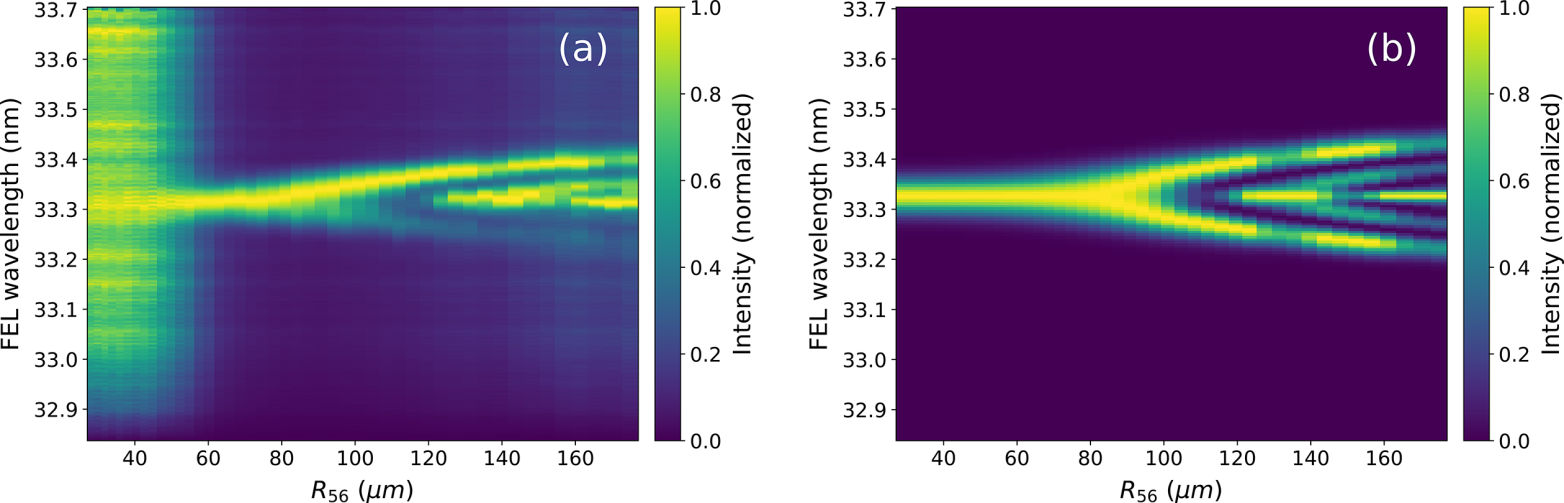
Normalized FEL spectra as a function of the dispersive section for the experiment (*a*) and for the model with fitted parameters (*b*).

The measured FEL spectra response to *R*_56_ [Fig. 6 ▸(*a*)] shows the characteristic bifurcation for a moderately chirped seed laser (Gauthier *et al.*, 2015 ▸), and the results are well reproduced by the model using the nominal electron beam parameters.

**Figure 7:**
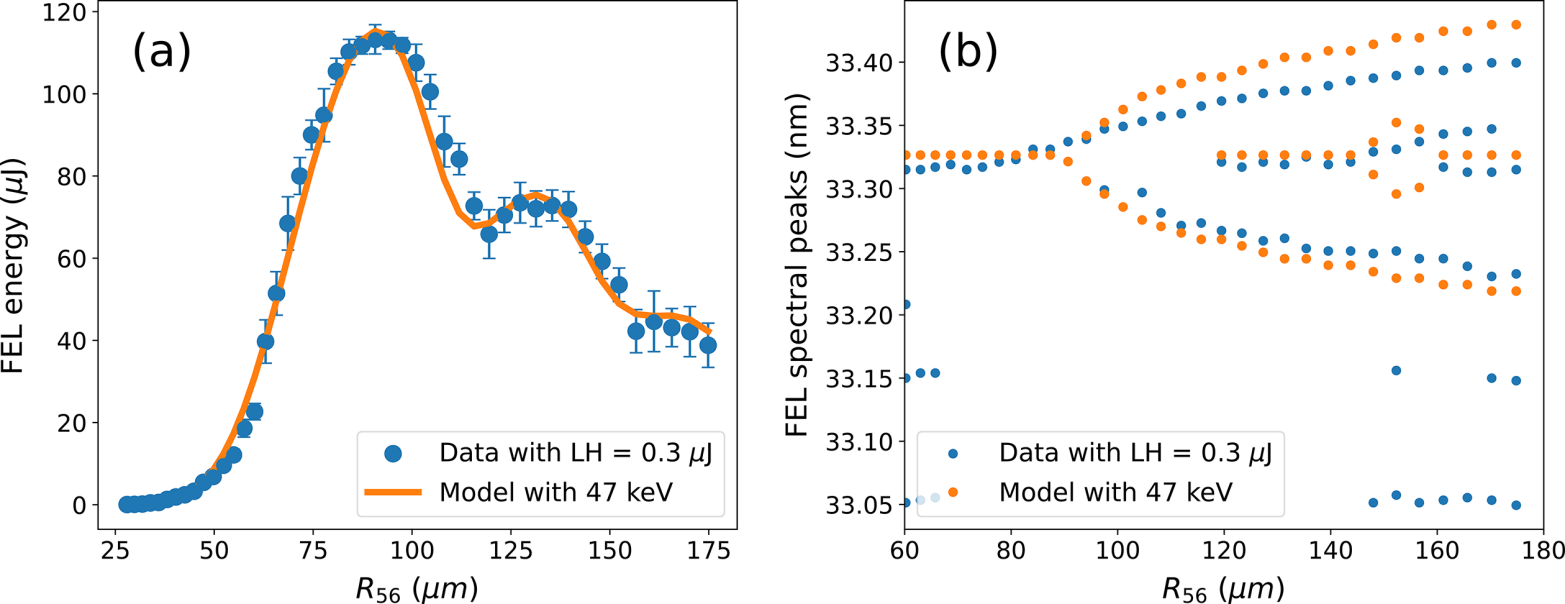
FEL energy and wavelengths of the FEL peaks as a function of the strength of the dispersive section. Blue curves refer to experimental data and orange to the model with the parameters obtained from the fitting.

Thanks to the use of normalized spectra, the agreement between the model and the experiments mainly depends on the seed laser energy modulation and residual chirp, which is directly connected to the seed laser pulse length. The agreement of the evolution of the positions of the FEL spectral peaks as a function of the dispersion [Fig. 7 ▸(*b*)] is an experimental validation for the seed laser pulse length measurement. More accurate measurements of all other parameters are done using the measured FEL pulse energy as a function of the chicane strength [Fig. 7 ▸(*a*)]. For the model data used in Fig. 7 ▸(*a*), the laser-induced energy modulation Δ_γ_ has been finely adjusted via small changes of the seed laser waist at the undulator [see equation (2)] while keeping the energy constant to the measured value. This, together with the variations to the current of the electron beam, and the slice energy spread of the beam, have allowed the best agreement to be found between the experimental curve (blue) and that from the model (orange). All other parameters are set to the measured or nominal ones.

For the analyzed scan, the model suggests that the beam has a peak current of 520 ± 5 A, a slice energy spread of 47 ± 1 keV and a waist (1/*e*^2^) for the seed of 1.10 ± 0.02 mm, which is slightly larger than the one estimated from the virtual undulator diagnostic (1.3 mm), but the difference is well within the expected values given the 30% uncertainty coming from the mode quality reported above.

### 4.2. Variation of slice energy spread

**Figure 8:**
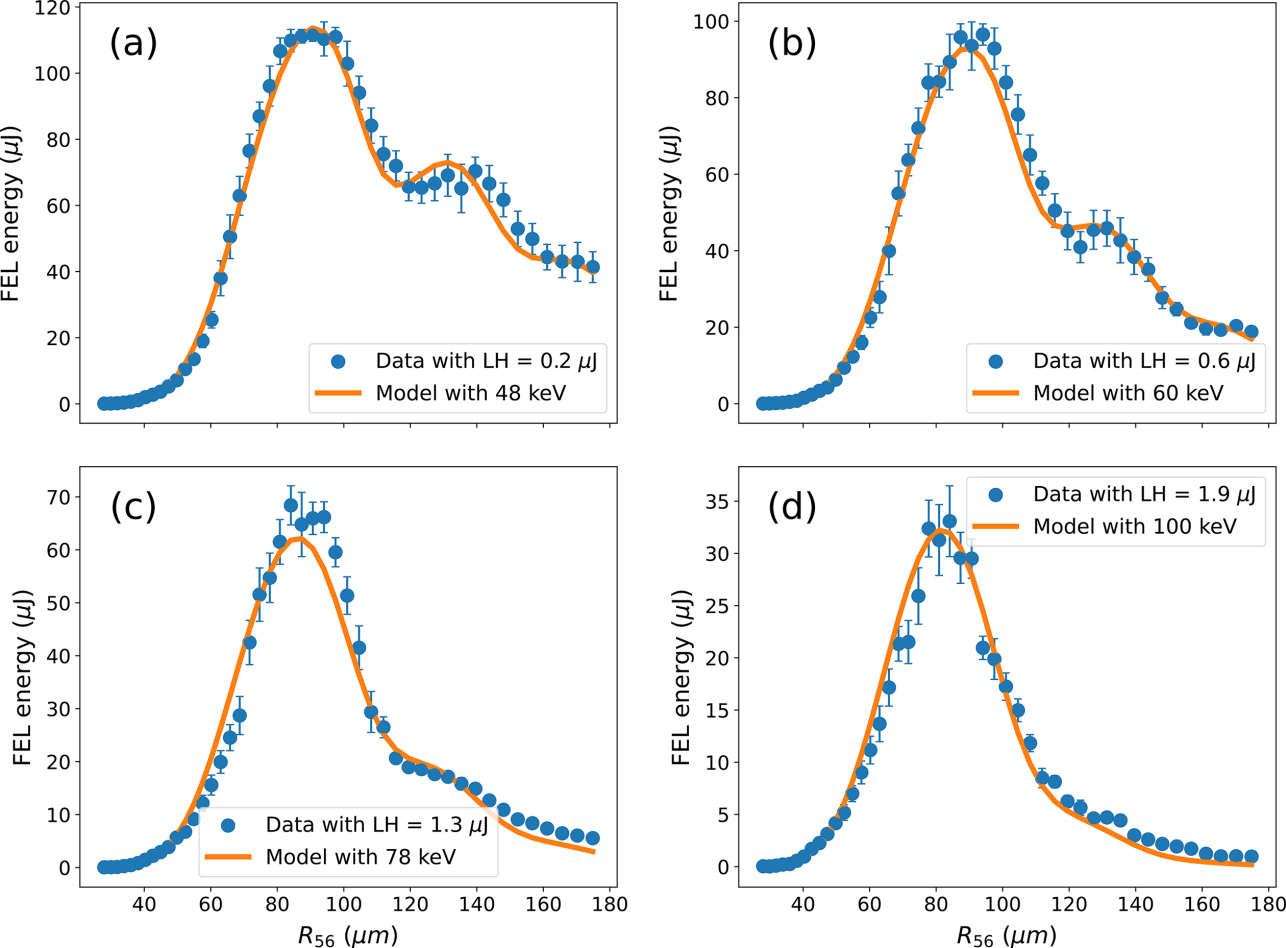
FEL energy as a function of the strength of the dispersive section (blue curves refer to experimental data and orange to the model) for different values of the laser heater energy. The used laser heater energy and retrieved slice energy spread by the model are reported in the figure labels.

To validate the method, we can repeat measurements for different values of the electron beam slice energy spread. This can be easily done by changing the laser heater energy per pulse (Spampinati *et al.*, 2014 ▸). Fig. 8 ▸ reports the FEL intensity as a function of the dispersion strength for lower [Fig. 8 ▸(*a*)] and higher [Figs. 8 ▸(*b*), 8(*c*) and 8(*d*)] laser heater intensity. The model data (orange curves) have been obtained with the parameters used for Fig. 7 ▸ with the only change being the electron beam slice energy spread to match the experimental results. It is worth noting that results in Fig. 8 ▸(*a*), despite referring to a lower value of the laser heater intensity with respect to Fig. 7 ▸, indicate a very similar value for the electron beam slice energy spread. This is the result of the micro-bunching instability (Venturini, 2007 ▸) that develops if not enough laser heater intensity is used as discussed below.

**Figure 9:**
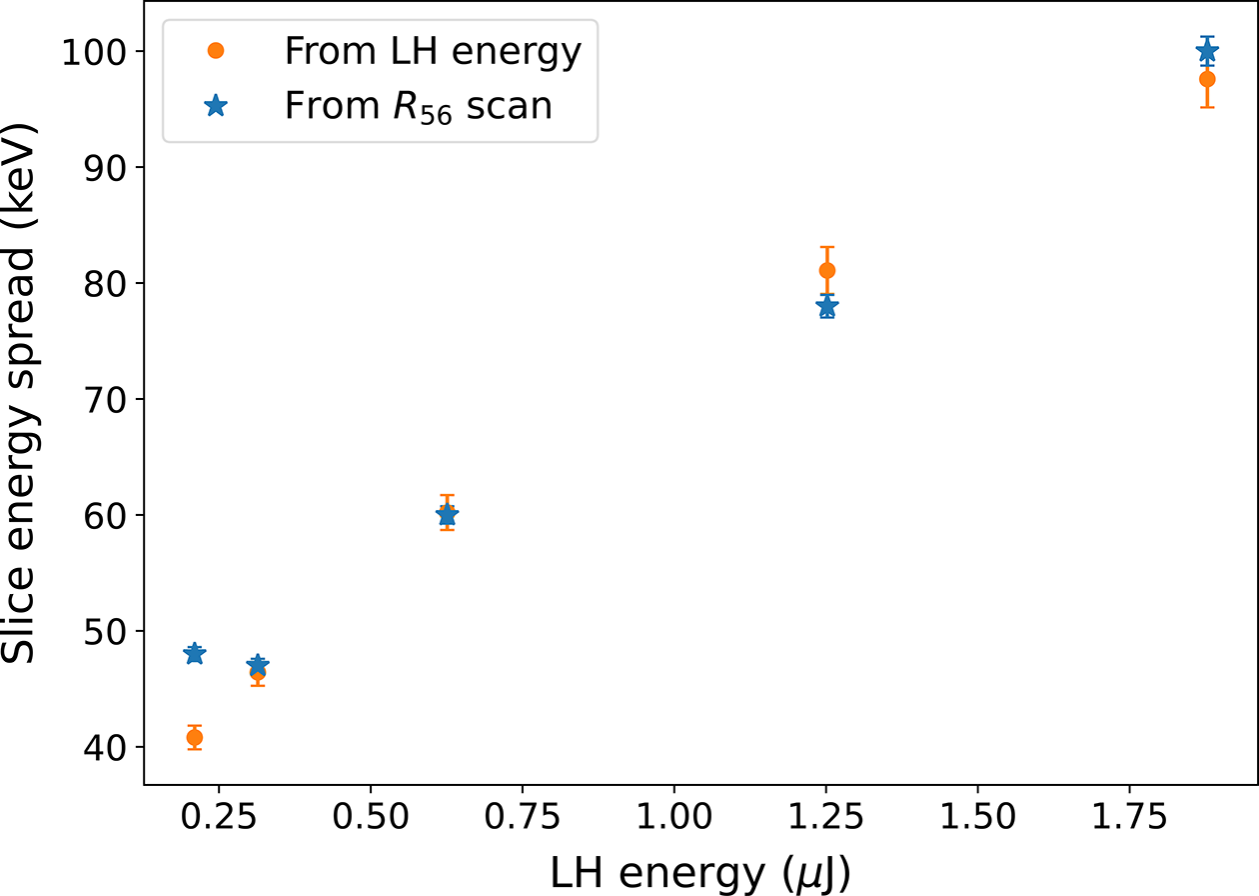
Comparison of the slice energy spread obtained with the reported method and that predicted from the intensity of the laser heater.

The method used for determining the electron beam parameters shows extraordinary robustness and high accuracy in determining the electron beam slice energy spread. The measured slice energy spread can be compared with the one predicted by means of equations (2) and (3) using the parameters for the beam, the laser and the undulator at the laser heater. By using the nominal and measured parameters and assuming a compression of the beam in the linac by a factor of ten, which is the nominal one for FERMI, we obtain the theoretical beam slice energy spread data reported in Fig. 9 ▸ (blue stars) that are very well superposed to those estimated from the setting of the laser heater (orange stars). For the theoretical values of the beam slice energy spread we assumed an uncertainty in the exact value of the laser heater energy of 10% coming from some unknown in the laser transport optics giving an ∼5% uncertainty on the induced slice energy spread.

It can be noted that for the lowest shown value of the laser heater energy the slice energy spread obtained from the experimental data is significantly different from that predicted by the laser heater settings, being also larger than the value obtained for a larger laser heater energy.

This result is very reproducible and has to be attributed to the well known problem of micro-bunching instability (Venturini, 2007 ▸) that the beam develops in the linac. For a beam not sufficiently heated by the laser heater (Huang *et al.*, 2010 ▸), the gain of the micro-bunching introduces an extra-slice energy spread that can overcome the original values. This fact is confirmed by independent measurements, not reported here, showing that for low values of the laser heater energy the FEL spectrum shows some sidebands resulting from the structures introduced into the beam by the micro-bunching.

## 5. Conclusions

With this work, we have reported a new, efficient method for retrieving the slice parameters for the peak current, laser-induced energy modulation and slice energy spread of the electron beam in seeded FELs, which is essential for properly setting the FEL. The method has been validated through experiments performed at FERMI under various conditions, demonstrating high-quality data retrieval and good reliability. Due to its achieved level of accuracy and the fact that it does not require additional hardware beyond what is already available in any externally seeded FEL, this method can become an important tool for all existing and future seeded FELs (Spezzani *et al.*, 2024 ▸; Allaria *et al.*, 2021 ▸; Zhentang *et al.*, 2019 ▸).

## Figures and Tables

**Table 1:** Nominal FERMI electron beam parameters during the experiment

Parameter	Value	Units	Accuracy/stability (%)
Charge	500	pC	3
Peak current	500	A	10
Slice emittance	1.0	mm mrad	20
Energy	1.369	GeV	0.01
Slice energy spread	50–150	keV	
Beam size	50–80	µm	10
Energy chirp	2–3	MeV ps^−1^	0.1
Length (FWHM)	1.0	ps	10

**Table 2:** Parameters for the seed laser used during the measurements [full width at half-maximum (FWHM) values are used for width and length]

Parameters	Value	Units	Accuracy/stability (%)
Wavelength	266.0	nm	0.1
Bandwidth	1.2	nm	5
Pulse length	180	fs	10
Energy	20	µJ	10
Spot size (1/*e*^2^)	950	µm	30

**Table 3:** Values and accuracy of the settings for the FEL devices

Parameters	Value	Units	Accuracy/stability (%)
Modulator1 period	30 × 10.0	mm	–
Modulator1 (K)	3–6		0.1
Modulator2 period	14 × 11.3	mm	–
Modulator2 (K)	3–6		0.1
Radiator period	3 × 42 × 55.0	mm	0.1
Radiator (K)	1–2.5		0.1
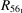 dispersion	0–10	mm	5
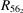 dispersion	0–200	µm	0.5
